# A comprehensive review of *Tripterygium wilfordii* hook. f. in the treatment of rheumatic and autoimmune diseases: Bioactive compounds, mechanisms of action, and future directions

**DOI:** 10.3389/fphar.2023.1282610

**Published:** 2023-11-01

**Authors:** Yu Shan, Jianan Zhao, Kai Wei, Ping Jiang, Lingxia Xu, Cen Chang, Linshuai Xu, Yiming Shi, Yixin Zheng, Yanqin Bian, Mi Zhou, Steven J. Schrodi, Shicheng Guo, Dongyi He

**Affiliations:** ^1^ Department of Rheumatology, Shanghai Guanghua Hospital, Shanghai University of Traditional Chinese Medicine, Shanghai, China; ^2^ Guanghua Clinical Medical College, Shanghai University of Traditional Chinese Medicine, Shanghai, China; ^3^ Institute of Arthritis Research in Integrative Medicine, Shanghai Academy of Traditional Chinese Medicine, Shanghai, China; ^4^ Arthritis Institute of Integrated Traditional and Western Medicine, Shanghai Chinese Medicine Research Institute, Shanghai, China; ^5^ Computation and Informatics in Biology and Medicine, University of Wisconsin-Madison, Madison, WI. United States; ^6^ Department of Medical Genetics, School of Medicine and Public Health, University of Wisconsin-Madison, Madison, WI, United States

**Keywords:** rheumatic and autoimmune diseases, Tripterygium wilfordii Hook F, antiinflammatory, immune-modulating, immunosuppressive effects

## Abstract

Rheumatic and autoimmune diseases are a group of immune system-related disorders wherein the immune system mistakenly attacks and damages the body’s tissues and organs. This excessive immune response leads to inflammation, tissue damage, and functional impairment. Therapeutic approaches typically involve medications that regulate immune responses, reduce inflammation, alleviate symptoms, and target specific damaged organs. *Tripterygium wilfordii* Hook. f., a traditional Chinese medicinal plant, has been widely studied in recent years for its application in the treatment of autoimmune diseases, including rheumatoid arthritis, systemic lupus erythematosus, and multiple sclerosis. Numerous studies have shown that preparations of *Tripterygium wilfordii* have anti-inflammatory, immunomodulatory, and immunosuppressive effects, which effectively improve the symptoms and quality of life of patients with autoimmune diseases, whereas the active metabolites of *T. wilfordii* have been demonstrated to inhibit immune cell activation, regulate the production of inflammatory factors, and modulate the immune system. However, although these effects contribute to reductions in inflammatory responses and the suppression of autoimmune reactions, as well as minimize tissue and organ damage, the underlying mechanisms of action require further investigation. Moreover, despite the efficacy of *T. wilfordii* in the treatment of autoimmune diseases, its toxicity and side effects, including its potential hepatotoxicity and nephrotoxicity, warrant a thorough assessment. Furthermore, to maximize the therapeutic benefits of this plant in the treatment of autoimmune diseases and enable more patients to utilize these benefits, efforts should be made to strengthen the regulation and standardized use of *T. wilfordii.*

## 1 Introduction

Rheumatic and autoimmune diseases are chronic conditions that affect millions of people worldwide. These diseases are characterized by inflammation, pain, and damage to the affected organs and tissues, and thus significantly affect the quality of life of patients (Schwenzer et al., 2010; Ma et al., 2020). Several treatment modalities are currently available for these diseases, including the administration of non-steroidal anti-inflammatory drugs, disease-modifying antirheumatic drugs, and biological agents (Tsitrouli et al., 2021). However, these therapeutic approaches do not always fully satisfy the clinical needs of patients and may be associated with potential side effects. Additionally, patients may exhibit an inadequate response to these strategies, and the occurrence of adverse events, coupled with their high cost, imposes a substantial burden on clinical healthcare (Shi et al., 2020). Consequently, there is a compelling need to identify effective novel drugs that could be applied in the treatment of rheumatic and autoimmune diseases.

*Tripterygium wilfordii* Hook. f. is a herb used in traditional Chinese medicine that was first recorded in the Compendium of Materia Medica (Ben Cao Gang Mu Shi Yi), and has been widely used to treat various diseases, including rheumatic and autoimmune disorders, for centuries (Jia et al., 2021). *T. wilfordii* is used as a herbal medicine in traditional Chinese medicine to treat joint pain, promote blood circulation, relieve rheumatism, and alleviate pain and swelling (Shen et al., 2021). In recent decades, the assessment of the therapeutic potential of *T. wilfordii* has attracted increased interest, which has led to extensive research on its pharmacological properties and potential clinical applications. Owing to its strong anti-inflammatory and immunomodulatory properties, *T. wilfordii* is considered a promising candidate for the treatment of rheumatic diseases and autoimmune disorders (Hou et al., 2017).

Preliminary clinical studies have indicated that *T. wilfordii* and its active metabolites have beneficial effects in the treatment of a range of rheumatic and autoimmune disorders, including rheumatoid arthritis (RA), systemic lupus erythematosus (SLE), and psoriasis (Gonçalves et al., 2020). For example, in a mouse model of collagen-induced arthritis, treatment with triptolide was found to significantly reduce disease severity and inhibit pro-inflammatory cytokine production and immune cell activation (Yang et al., 2016). Similarly, in a mouse model of lupus nephritis, treatment with celastrol was demonstrated to significantly reduce disease severity and reduce the levels of autoantibodies and pro-inflammatory cytokines (Chen et al., 2022). Clinical studies have also reported promising outcomes with the use of *T. wilfordii* for the treatment of rheumatic diseases and autoimmune disorders (Yu et al., 2020). For example, in a randomized, double-blind, placebo-controlled trial, patients with RA who received *T. wilfordii* extract showed significant improvements in disease severity and joint function compared with those who received a placebo (Jiang et al., 2017). In a similar trial, patients with psoriasis who received a *T. wilfordii* extract showed significant improvements in disease severity and skin symptoms compared with those who received a placebo (Lv et al., 2018).

Considering its potent anti-inflammatory and immunomodulatory properties, *T. wilfordii* has shown significant potential as a therapeutic agent for the treatment of rheumatic diseases and autoimmune disorders (Song CY. et al., 2020). Although further research is needed to gain a more comprehensive understanding of the mechanisms underlying the effects of *T. wilfordii* and its active metabolites, evidence obtained to date indicates that preparations of this plant may offer a safe and effective alternative to currently available treatment modalities (Lv et al., 2018). However, more studies will be required to determine the optimal dosages and treatment regimens for *T. wilfordii,* as well as to identify any potential side effects or drug interactions. In this review, we discuss the evidence supporting the use of *T. wilfordii* in the treatment of rheumatic diseases and autoimmune disorders, with a focus on key findings from preclinical and clinical studies.

## 2 Historical uses of *Tripterygium* *wilfordii* in traditional medicine

**FIGURE 1:**
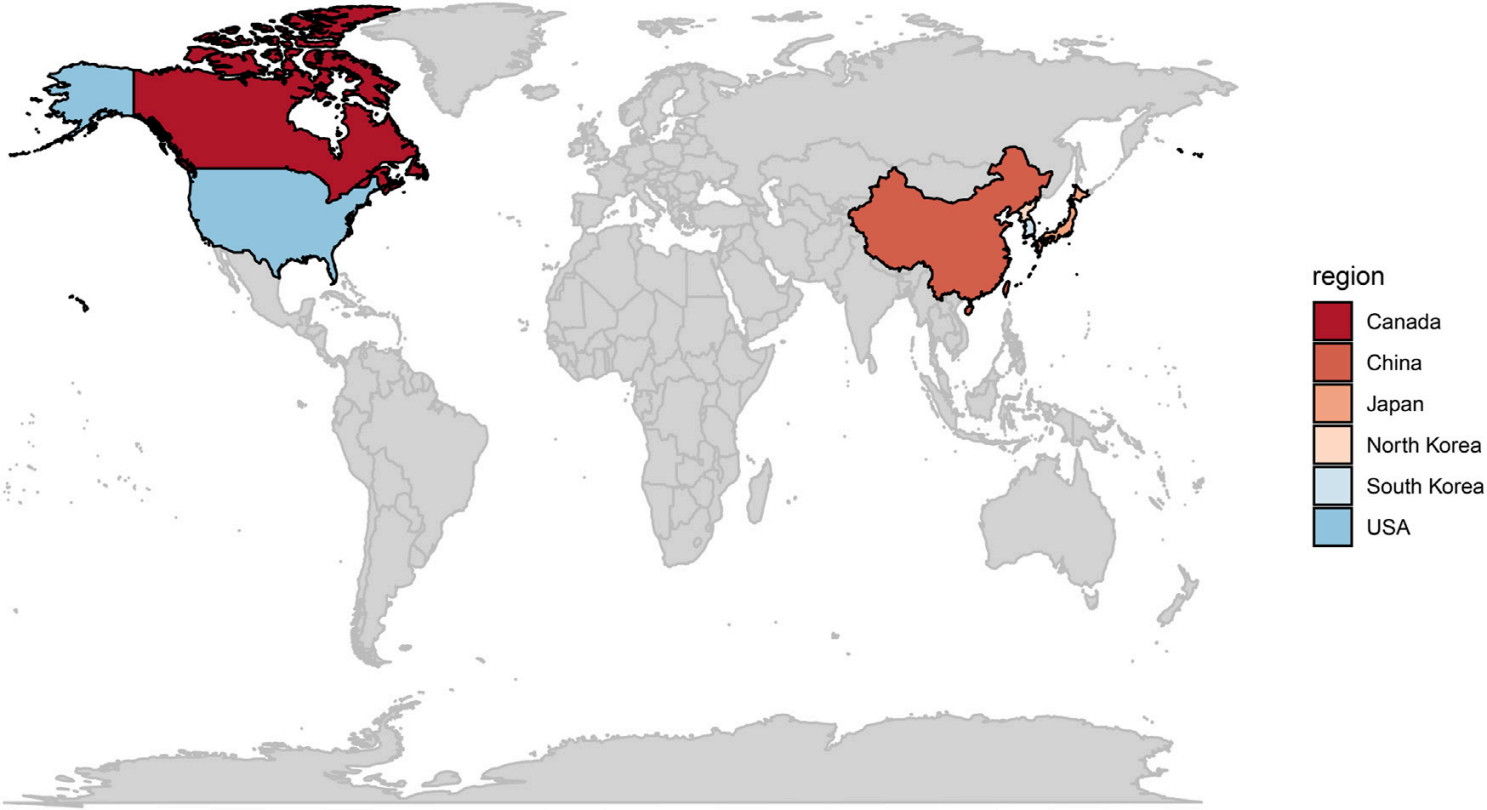
Natural global distribution of *Tripterygium wilfordii*.

*T. wilfordii* has a long history of use in traditional medicine. Natural populations of this vine are widely distributed in moist regions of the Northern Hemisphere, including China, Japan, North and South Korea, the United States, and Canada, with a total of 1,219 locations confirmed globally (Figure 1) (Cheng et al., 2021). In traditional Chinese medicine, *T. wilfordii* is considered a potent herbal medicine, which can be used to treat a range of diseases and disorders (Lu et al., 2022). The medicinal use of *T. wilfordii* can be traced back to the Han Dynasty (220 BC), at which time it was used to treat various ailments such as gout, edemas, and fever. Since then, the plant has also been used to treat rheumatic diseases and promote blood circulation. During the Tang Dynasty (618–907 AD), the use of *T. wilfordii* became even more widespread and was used to treat a wider range of conditions, including inflammation, swelling, and joint pain (Lu et al., 2022), and the use of this plant in traditional medicine has continued to the present day. Currently, *T. wilfordii* is commonly administered to treat RA, SLE, and other autoimmune diseases, and its anticancer properties have been recognized (Xue et al., 2012). Furthermore, the traditional use of *T. wilfordii* has been documented in South Korea and Japan. In South Korea, this plant is referred to as “jeokseong” and has been used in the treatment of RA, while in Japan, *T. wilfordii* is known as “raiji,” and is recognized not only for its therapeutic properties in RA management (Cheng et al., 2021) but also for its use in the treatment of inflammatory diseases, such as Crohn’s disease and ulcerative colitis (Wang et al., 2016).

## 3 Chemical composition of *Tripterygium wilfordii*

Recently, considering its potential use in the treatment of rheumatic and autoimmune diseases, *T. wilfordii* has attracted the attention of the conventional medical community (Oliner et al., 2016). To date, over 500 metabolites have been isolated and identified from *T. wilfordii*, including sesquiterpenes, diterpenes, triterpenes, alkaloids, flavonoids, lignans, and glycosides. Among these metabolites, notable compounds, such as triptolide, celastrol, and wilforlide, are believed to contribute to the diverse pharmacological activities of *T. wilfordii*. The identification and characterization of such compounds can provide valuable insights into the chemical composition and potential therapeutic properties of this plant (Luo et al., 2019; Xu et al., 2019). The following subsections provide an overview of the major metabolites isolated from *T. wilfordii* in modern medicine and their current status in terms of mechanistic research. (Table 1)

**TABLE 1:** Chemical composition of *Tripterygium wilfordii* Hook F.

Name	Molecular formula	Molecular structure	Properties	References
**Triptolide**	C_20_H_24_O_6_	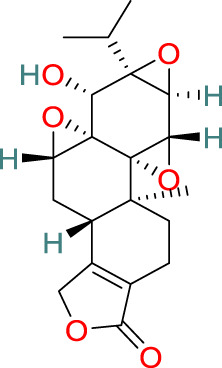	Anti-inflammatory, immunosuppressive, anti-cancer	Kupchan et al. (1972), Gao et al. (2021), Tong et al. (2021), Zhao et al. (2013), Wen et al. (2012), Liu et al. (2013a)
**Celastrol**	C_29_H_38_O_4_	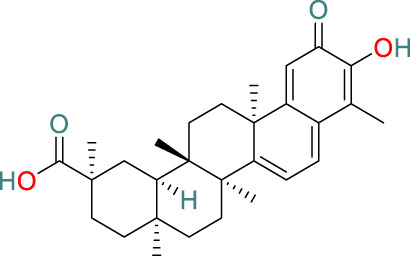	Anti-cancer, antioxidant, anti-obesity, neuroprotective and immunosuppressive	Hou et al. (2020), Xu et al. (2021), Hernandes et al. (2017), Liu et al. (2022), Song et al. (2020b)
**Wilforlide A**	C_30_H_46_O_3_	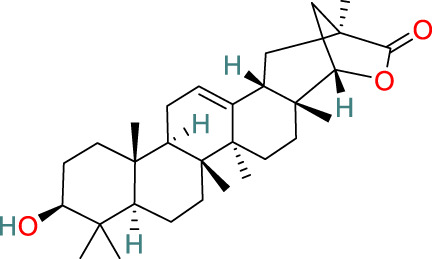	Anti-inflammatory, immunosuppressive	Xue et al. (2010), Wang et al. (2018), Cao et al. (2022), Mao et al. (2021)
**Sesquiterpenes**	C_15_H_18_O_3_	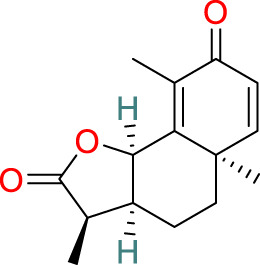	Anti-cancer, anti-inflammatory, anti-bacterial, anti-parasitic, insecticidal, anti-viral	Miller and Allemann, (2012), Chen et al. (2020)
**Diterpenes**	C_20_H_32_O_3_	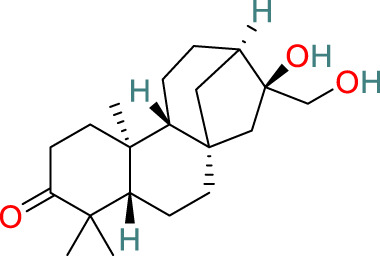	Immunomodulation, metabolic modulation, anti-cancer, anti-viral, anti-depressant	Qu et al. (2022), Yuan et al. (2019), Guo et al. (2016)
**Triterpenes**	C_47_H_80_O_17_	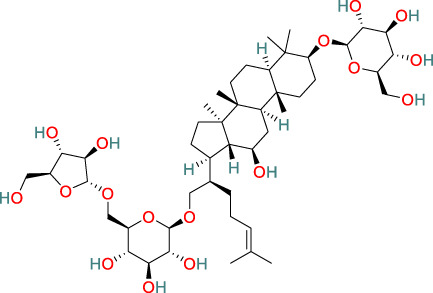	Anti-cancer, anti-inflammatory, cardioprotective, anti-viral, anti-fibrotic, immunosuppressive, regulates metabolic dysfunction	Hu et al. (2017), Chang et al. (2016), Ghante and Jamkhande, (2019), Timilsina et al. (2016)
**Alkaloids**	C_23_H_29_N_3_O_2_	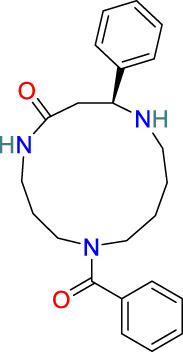	Anti-inflammatory, anti-viral, immunosuppressive and insecticidal activity	Fang et al. (2012), Horiuch et al. (2006), Tang et al. (2017)
**Glycosides**	C_29_H_44_O_12_	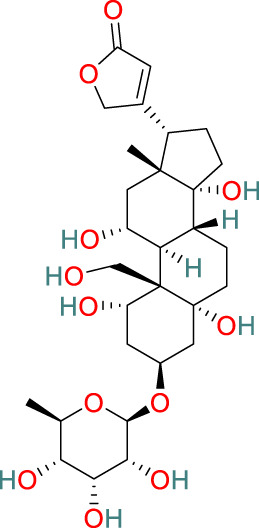	Anti-inflammatory, immunosuppressive	Zhang and Chen, (2017), Zhang et al. (2021), Yang et al. (2020)
**LLDT-8**	C_20_H_24_O_7_	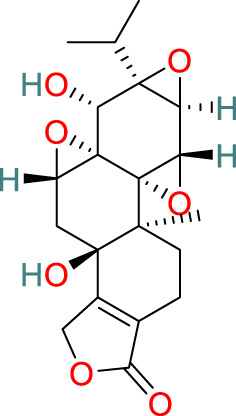	Anti-inflammatory, immunosuppressive	Su et al. (2017), Cui et al. (2019), Zhou et al. (2021), Guo et al. (2019), Ma et al. (2017), Ziaei and Halaby, (2016), Ibrahim et al. (2021), Dong et al. (2019)

### 3.1 Triptolide

Triptolide, one of the key active compounds identified in *T. wilfordii*, is primarily obtained through extraction and isolation from various medicinal plants. First isolated by Kupchan et al., in 1972, triptolide has been established to have significant anti-leukemic activity (Kupchan et al., 1972) in addition to other important biological activities, including prominent anti-inflammatory, immunosuppressive, and anticancer effects (Gao et al., 2021). Triptolide exerts its anticancer effects by modulating different signaling pathways and gene expression at multiple levels, as well as by interacting with miRNAs. Specifically, these therapeutic effects are mediated via the modulation of cell apoptosis, senescence, proliferation, invasion, migration, and angiogenesis (Tong et al., 2021). For example, triptolide has been established to induce apoptosis in prostate cancer cells through the activation of caspases and poly-ADP-ribose polymerase cleavage (Zhao et al., 2013). Moreover, it inhibits nuclear factor kappa-B (NF-κB) activation and downregulates the expression of toll-like receptor 4 (TLR4) (Wen et al., 2012). Reportedly, triptolide can also contribute to the prevention of bone deterioration by reducing the number of osteoclasts in inflamed joints, reducing the expression of receptor activator of NF-κB (RANK) and its ligand RANKL, enhancing the expression of osteoprotegerin (OPG) at the mRNA and protein levels, and lowering the ratio of RANKL to OPG in the serum and inflamed joints of mice with collagen-induced arthritis (Liu C. et al., 2013).

### 3.2 Celastrol

The pentacyclic triterpenoid celastrol has been identified as a major bioactive metabolite of *T. wilfordii* that has diverse biological properties, including antitumor, antioxidant, anti-obesity, neuroprotective, and immunosuppressive effects (Hou et al., 2020; Xu et al., 2021). Moreover, celastrol has been identified as a natural proteasome inhibitor, the activity of which accordingly leads to an accumulation of ubiquitinated proteins and proteasome substrates, such as NF-κB (IκBα), Bax, and p27, and induces the ubiquitination of orphan nuclear receptor NR4A1 (Nur77) through its Lys63 linkage (Hernandes et al., 2017). Under inflammatory conditions, ubiquitinated Nur77 accumulates within the mitochondria, thereby rendering these organelles sensitive to autophagy. This event involves an interaction between Nur77 and p62/SQSTM1, thereby contributing to an alleviation of inflammation (Liu et al., 2022). Celastrol has also been shown to target multiple signaling pathways, including those of NF-κB, endoplasmic reticulum Ca^2+^-ATPase, myeloid differentiation factor 2, TLR4, pro-inflammatory chemokines, DNA damage, cell cycle arrest, and apoptosis (Song X. et al., 2020).

### 3.3 Wilforlide A

Wilforlide A is a triterpene isolated from *T. wilfordii* that can serve as a quality control standard for tripterygium glycosides, a class of drugs derived from *T. wilfordii.* Wilforlide A also exhibits potent anti-inflammatory and immunosuppressive activities (Xue et al., 2010), and has been used alone as a traditional botanical drug for the treatment of autoimmune diseases and is widely used to treat conditions such as RA, SLE, and psoriasis (Wang et al., 2018). Regarding its mechanism of action, it has been established that wilforlide A inhibits the secretion of pro-inflammatory cytokines (MCP1, GM-CSF, and M-CSF) and the M1 biomarker inducible nitric oxide synthase in synovial cells (Cao et al., 2022). In *in vitro* experiments, wilforlide A was found to inhibit the lipopolysaccharide- and interferon-γ (IFN-γ)-induced upregulation of TLR4, degradation of IκBα, and activation of NF-κB p65 (Cao et al., 2022). Additionally, treatment with the TLR4 inhibitor TAK242 has been demonstrated to have inhibitory effects on M1 polarization similar to those of wilforlide, and the combined administration of TAK242 and wilforlide A has been found to enhance these inhibitory effects (Mao et al., 2021).

### 3.4 Sesquiterpenes

The sesquiterpenes isolated from *T. wilfordii* are the products of the metabolism of approximately 300 different C_15_ isoprenoid precursors, which are synthesized from the single substrate farnesyl diphosphate by sesquiterpene synthases (Miller and Allemann, 2012). Two novel sesquiterpenes, namely, 1β,2α,6α,8β,15-pentaacetoxy-9α-benzoyloxy-β-dihydroagarofuran and 1β,2β,6α,15-tetraacetoxy-9β-benzoyloxy-β-dihydroagarofuran, have recently been discovered in *T. wilfordii* and found to exhibit inhibitory effects against A549 human lung cancer cells, human osteosarcoma cells, and human breast cancer cell lines. Moreover, sesquiterpenes have been reported to have a range of pharmacological properties, including antitumor, anti-inflammatory, antimicrobial, antiparasitic, insecticidal, and antiviral activities (Chen et al., 2020).

### 3.5 Diterpenes

Diterpenes are among the major active metabolites of *T. wilfordii* and can be further classified as abietane, tigliane, and ingenane diterpenes (Qu et al., 2022). Among these, triptolide was first isolated from *T. wilfordii* in 1972 by Kupchan, and exhibits potent pharmacological activities against various immune disorders, cancers, viruses, fibrosis, asthma, depression, and metabolic imbalances (Guo et al., 2016; Yuan et al., 2019).

### 3.6 Triterpenes

The triterpenes present in *T. wilfordii* are primarily classified into friedelanes, oleananes, and ursanes (Chang et al., 2016; Hu et al., 2017). Pentacyclic triterpenes are a class of representative plant compounds produced as secondary metabolites, which have been shown to possess diverse biological activities, including anticancer, anti-inflammatory, cardioprotective, and antidiabetic effects (Ghante and Jamkhande, 2019). Bevirimat, a triterpene compound extracted from *T. wilfordii*, has significant antiviral, antifibrotic, immunosuppressive, metabolic dysfunction-regulating, and spermicidal activities (Song X. et al., 2020). Notably, it is the only triterpene derivative with anti-human immunodeficiency virus properties that has been assessed to date in Phase II clinical trials (Timilsina et al., 2016).

### 3.7 Alkaloids

The alkaloids present in *T. wilfordii* can be classified into two main categories, sesquiterpenes and indole alkaloids, both of which are characterized by prominent anti-inflammatory, antiviral, immunosuppressive, and insecticidal properties (Fang et al., 2012). To date, four indole alkaloids have been isolated from *T. wilfordii*, namely, celabazine, celacinnine, celafurine, and celallocinnine (Horiuch et al., 2006), and the findings of relevant studies have indicated that celacinnine and celallocinnine interact with matrix metalloproteinase-9, thereby contributing to an alleviation of skin inflammation (Tang et al., 2017).

### 3.8 Glycosides

Glycosides extracted from the roots of *T. wilfordii* have been demonstrated to exert anti-inflammatory and immunosuppressive effects by inhibiting the release of inflammatory cytokines such as interleukin (IL)-6, IL-1β, and tumor necrosis factor-alpha (TNF-α) (Zhang and Chen, 2017). These glycosides have been used extensively in the treatment of inflammatory diseases (Zhang et al., 2021). Mechanistically, they can reduce the activity of NF-κB, suppress the gene expression of cyclooxygenase-2 and M1 biomarker inducible nitric oxide synthase, and reduce the production of prostaglandin E2 and nitric oxide, thereby exerting anti-inflammatory effects (Yang et al., 2020).

### 3.9 LLDT-8

5R-Hydroxytriptolide (LLDT-8) is a novel analog of *T. wilfordii*-derived triptolide characterized by low cytotoxicity and high immunosuppressive activity (Su et al., 2017), and numerous *in vitro* and *in vivo* studies have demonstrated the significant anti-inflammatory and immunosuppressive properties of this compound. LLDT-8 has been approved by the China Food and Drug Administration as an immunosuppressive drug in clinical trials for RA treatment (Cui et al., 2019), and is currently being investigated as a low-toxicity immunosuppressive agent in Phase I clinical trials for RA in China. The findings of early studies indicated that the activity of LLDT-8 may be associated with anti-inflammatory, antioxidant, and cytokine effects and that it can prevent bleomycin-induced pulmonary fibrosis in mice (Zhou et al., 2021). Recently, the first long non-coding RNA-transcription factor-mRNA co-expression network was constructed to further elucidate the changes in genome-wide long non-coding RNA and mRNA expression before and after LLDT-8 treatment, which indicated that long non-coding RNAs could serve as biomarkers and targets for LLDT-8 drug development (Guo et al., 2019). Additionally, LLDT-8 has been found to have potent anti-inflammatory effects, including the amelioration of anti-glomerular basement membrane glomerulonephritis by modulating the Fcγ signaling pathway and suppression of renal chemokine expression to inhibit immune cell infiltration, thereby ameliorating lupus nephritis (Ma et al., 2017). The protective effects of LLDT-8 include the elimination of activated T cells by promoting the expression of the pro-apoptotic gene signal transducer and activator of signal transducer and activator of transcription 1 and interferon regulatory factor-1 in the spleen (Ziaei and Halaby, 2016). LLDT-8 has also been established to reduce the production of IFN-γ, IL-2, and TNF-α by peripheral blood mononuclear cells (Ibrahim et al., 2021), and has therapeutic potential for the treatment of neurodegenerative diseases by effectively inhibiting pro-inflammatory cytokines (TNF-α and IL-1β) and suppressing the NF-κB signaling pathway, thereby reducing neuroinflammation (Ziaei and Halaby, 2016). Furthermore, LLDT-8 has been demonstrated to reduce the levels of serum alanine aminotransferase and aspartate aminotransferase, liver balloon cell formation, and macrovesicular steatosis, thereby preventing liver injury (Dong et al., 2019). LLDT-8 also regulates the expression of stearoyl-CoA desaturase-1 and peroxisome proliferator-activated receptor alpha, which contribute to promoting significant degradation of lipids and suppression of lipid synthesis (Gao et al., 2021).

## 4 *Tripterygium wilfordii* in the treatment of RA

The earliest studies on *T. wilfordii* were conducted in the 1970s, during which its efficacy in the treatment of RA was initially discovered. Since then, numerous studies have investigated the therapeutic effects of *T. wilfordii* in the treatment of other rheumatic diseases and autoimmune disorders, including SLE, psoriasis, and multiple sclerosis (Table 2) (Xu et al., 2008). A meta-analysis of randomized controlled trials conducted to assess the efficacy of *T. wilfordii* in the treatment of RA found that the use of *T. wilfordii* glycoside tablets alone could reduce the number of tender and swollen joints in patients with RA, with an efficacy comparable to that of methotrexate (MTX). Furthermore, a combination of *T. wilfordii* glycoside tablets and MTX was found to be superior to the administration of MTX alone in improving the clinical symptoms of patients with RA (Zhou et al., 2018). Similarly, a further systematic review and network meta-analysis evaluating the efficacy and safety of *T. wilfordii* in treating RA found that the glycosides of this plant administered in combination with MTX may have been the optimal treatment of choice based on an assessment of the ACR20 response. Moreover, among the different treatments assessed, the use of *T. wilfordii* glycosides alone ranked second only to the *T. wilfordii* glycoside + MTX combined treatment (Wang et al., 2022).

**TABLE 2:** Clinical trials on the active ingredients of *Tripterygium wilfordii* Hook F.

Name	Condition or disease	ClinicalTrials.gov number	Sponsor	Interventions	Primary outcomes	Phase
**Tripterygium Wilfordii Hook F**	IgA Nephropathy	NCT02187900	Second Xiangya Hospital of Central South University	TwHF/Mycophenolate mofetil (MMF)	1.Number of patients reaching remission	Phase 3
					2.Renal survival	
	Rheumatoid Arthritis	NCT04136262	Guang’anmen Hospital of China Academy of Chinese Medical Sciences	Tripterygium wilfordii Hook F (TwHF)/Methotrexate	1.Percentage of ACR20	Phase 3
					2. Percentage of ACR50	
					3. Percentage of ACR70	
					4. RAMRIS score	
	Human Immunodeficiency Virus	NCT01666990	Beijing 302 Hospital	TwHF/Placebo treatment	1.The total CD4 T cell counts compared with CD4 T cell counts at baseline 2.The CD38 expression on CD8 T cells	Phase 2
	Arthritis, Rheumatoid	NCT01613079	Peking Union Medical College Hospital	Tripterygium wilfordii Hook F/Methotrexate	1. The proportion of patients achieving ACR50	Phase 3
					2. Radiology outcome	
					3. DAS28	
					4. ACR20/70	
	Nephritis, Lupus	NCT01646736	Peking Union Medical College Hospital	Tripterygium wilfordii Hook F/Cyclophosphamide/GC	1. The proportion of patients achieving Complete Response (CR) and Partial Response (PR)	Phase 3
					2. Renal Function	
					3. Serum Albumin Level	
	Rheumatoid Arthritis	NCT03337815	Guang’anmen Hospital of China Academy of Chinese Medical Sciences	MTX and TwHF placebo/TwHF and MTX placebo	1.The change in Disease Activity Score (DAS28)	Phase 3
					2. The proportion of patients achieving ACR20/50/70	
	HIV	NCT02002286	LI Taisheng, Peking Union Medical College	Tripterygium Wilfordii Hook F extrac	1. Changes of T cell subsets and immune activation markers	Not Applicable
					2. Viral load	
					3. Number of participants with adverse events as a measure of safety and tolerability	
**Topical tripterygium gel**	Arthritis, Rheumatoid	NCT02818361	Guang’anmen Hospital of China Academy of Chinese Medical Sciences	Topical tripterygium gel/Placebo gel	1.Twenty percent improvement in the American College of Rheumatology criteria	Not Applicable
					2.Twenty percent improvement in the American College of Rheumatology criteria	
	Active Rheumatoid Arthritis	NCT01961505	Guang’anmen Hospital of China Academy of Chinese Medical Sciences	Topical compound Tripterygium/Placebo	1.ACR20 criteria	Not Applicable
					2. ACR50 criteria	
					3. 28-joint count Disease Activity Score	
**Tripterygium glycosides**	Inflammatory Bowel Diseases, Crohn’s Disease	NCT02044952	Zhu Weiming, Jinling Hospital, China	Tripterygium glycosides/Mesalazine	1.Therapeutic effect measured by Crohn’s Disease Activity Index (CDAI)	Phase 3
					2. The Side effects of Tripterygium wilfordii	
	Early Ankylosing Spondylitis	NCT00889694	Sun Yat-sen University	Tripterygium/Sulfasalazine/placebo	1. ASAS20	Phase 3
					2. BASDAI20/50/70	
	Lupus Nephritis	NCT00881309	Nanjing University School of Medicine	Tripterygium	1.Complete remission rate	Not Applicable
					2.Renal relapse	
					3. Partial remission	
	Diabetic Nephropathy	NCT00518362	Nanjing University School of Medicine	Tripterygium	1.To access the efficacy of TW compared to ARB in treatment of heavy proteinuria of diabetic nephropathy	Not Applicable

### 4.1 *Tripterygium wilfordii* and its derivatives inhibit RA-associated inflammation

**FIGURE 2:**
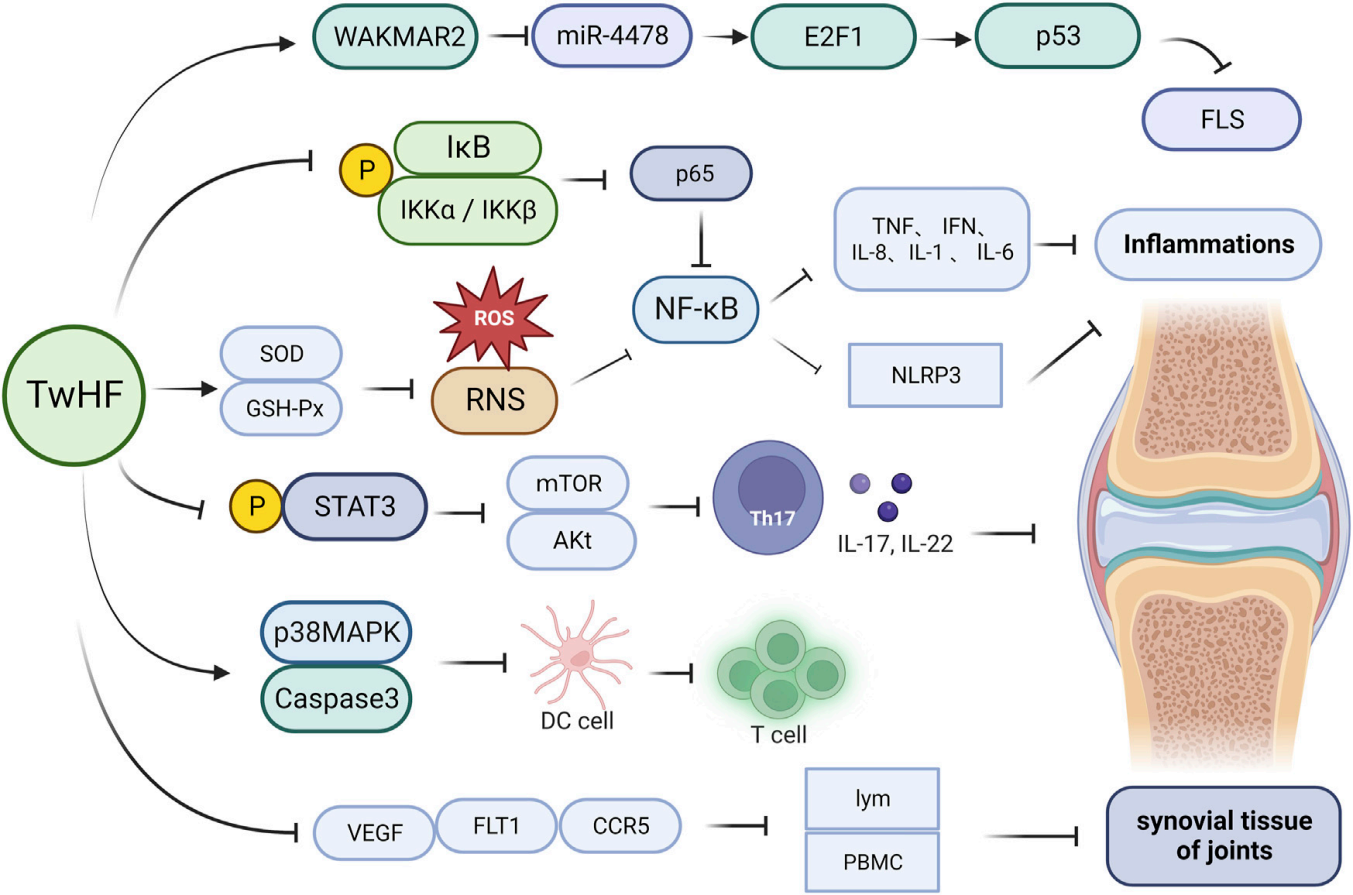
Mechanisms underlying the efficacy of *Tripterygium wilfordii* in the treatment of rheumatoid arthritis. Administration of *T. wilfordii* preparations can upregulate the levels of SOD and GSH-Px, thereby inducing the clearance of ROS and RNS within organisms. Additionally, *T. wilfordii* can significantly reduce the phosphorylation of IKKα/IKKβ and IκBα, subsequently inactivating p65. This in turn suppresses the NF-κB signal pathway, leading to a reduction in the release of cytokines, such as IL-1, IL-6, IL-8, TNF, IFN, and NLRP3, and thus achieving an anti-inflammatory effect. Furthermore, *T. wilfordii* can reduce the phosphorylation of STAT3, thereby reducing the expression of mTOR and Akt. This results in the metabolic disruption of Th17 cells, inhibiting their differentiation and reducing the release of inflammatory cytokines such as IL-17 and IL-22. Moreover, *T. wilfordii* induces DC apoptosis by activating p38 MAPK and caspase-3, thereby reducing the proliferation and differentiation of T cells*,* and suppresses the expression of VEGF, FLT1, and CCR5, leading to reduced lymphocyte and peripheral PBMCs of the synovium. This subsequently prevents the damage and deterioration of synovial tissue in the joints. Finally, *T. wilfordii* upregulates WAKMAR2 to inhibit the expression of miR-4478, subsequently upregulating E2F1 and p53 expression. This cascade leads to the suppression of FLS expression. (SOD, superoxide dismutase; GSH-Px, glutathione peroxidase; ROS, reactive oxygen species; RNS, reactive nitrogen species; NF-κB, nuclear factor kappa-B; TNF, tumor necrosis factor; IFN, interferon; NLRP3, NOD-like receptor protein 3; STAT3, signal transducer and activator of transcription 3; mTOR, mammalian target of rapamycin; AKt, protein kinase B; Th17, T helper cell 17; IL-17, interleukin 17; IL-22, interleukin 22; IL-1, interleukin 1; IL-6, interleukin 6; IL-8, interleukin 8; IκB, inhibitor of NF-κB; IKKα, inhibitor of kappa B kinase alpha; IKKβ, inhibitor of kappa B kinase beta; p65, nuclear factor kappa-B p65; p53, tumor suppressor protein; WAKMAR2, long non-coding RNA; miR-4478, microRNA 4,478; E2F1, E2F transcription factor 1; FLS, fibroblast-like synoviocyte; p38 MAPK, p38 mitogen-activated protein kinase; DC, dendritic cell; CCR5, chemokine receptor 5; PBMC, peripheral blood mononuclear cell; VEGF, vascular endothelial growth factor; FLT1, vascular endothelial growth factor receptor 1; lym, lymphocyte).

As discussed in previous sections, triptolide, celastrol, and wilforlide A have been shown to significantly inhibit inflammatory responses, with NF-κB activation in B cells being one of the primary targets (Urits et al., 2019). Targets downstream of NF-κB include pro-inflammatory cytokines such as TNF-β, which mediate the inflammatory response via NF-κB signaling (Lin, 2006). *T. wilfordii* glycosides, such as triptolide, celastrol, and wilforlide A, can exert anti-inflammatory effects by inhibiting the p56 subunit of the NF-κB signaling pathway, as well as by downregulating the expression of inflammatory factors such as TNF and IFN (Li et al., 2021). Studies have shown that NOD-like receptor protein 3 (NLRP3)-induced inflammation plays a key role in RA pathogenesis, and treatment with *T. wilfordii* has been demonstrated to inhibit activation of the NLRP3 inflammasome by blocking the NF-κB signaling pathway, thereby contributing reductions in joint swelling, arthritis index score, inflammatory cell infiltration, and synovial hyperplasia induced by complete Freund’s adjuvant in rats (Jing et al., 2023). Additionally, significant reductions in the secretion of IL-1β and IL-18 have been observed in rat serum and T helper 1 cell supernatant exposed to a *T. wilfordii* extract (Kim et al., 2019). Further research has revealed that celastrol can alleviate RA symptoms and suppress inflammation by inhibiting the reactive oxygen species (ROS)–NF–κB–NLRP3 axis. Moreover *T. wilfordii* glycosides have been established to upregulate the levels of superoxide dismutase and glutathione peroxidase, which induces the clearance of ROS and reactive nitrogen species, thereby reducing oxidative stress and inflammation (Figure 2) (Kim et al., 2019; Lv et al., 2022).

Celastrol has been found to significantly inhibit the protein and mRNA expression of the pro-inflammatory cytokines IL-6 and IL-8 and monocyte chemoattractant protein-1 induced by lipopolysaccharide in human retinal pigment epithelial and adult retinal pigment epithelial-19 cells (Tao et al., 2016). Further mechanistic studies have revealed that celastrol significantly reduces the phosphorylation of IKKα/IKKβ and IκBα, leading to the inactivation of p65, and thereby alleviating lipopolysaccharide-induced inflammation by inhibiting the NF-κB signaling pathway (Wang et al., 2017). Administration of *T. wilfordii* glycosides has also been shown to reduce the expression of the inflammatory factors IL-6, IL-8, NF-κB, and TNF-α (Liu et al., 2014), and promote an upregulation of the levels of IL-10 and CD4^+^CD25^+^ regulatory T cells (Tregs), increase the expression of forkhead box protein P3 (FoxP3), downregulate TNF-α and endothelin-1 levels to inhibit paw edema, and reduce the expression of high-mobility group protein 1 and IL-17 in the serum of rats with collagen-induced arthritis (Lei and Jian, 2012; Hao et al., 2022). In other studies, *T. wilfordii* glycosides have been demonstrated to inhibit the differentiation, maturation, and migration of immature dendritic cells, as well as the secretion of cytokines, thereby suppressing the activation of neutrophils and T cells through the transcriptional signal transducer and activator of STAT pathways. This leads to the downregulation of inducible cyclooxygenase-2, prostaglandins, and metalloproteinases, resulting in an attenuation of the inflammatory responses mediated by these cells (Tian et al., 2020; Kianmehr et al., 2023). Mitochondria play essential roles in cell death, autophagy, immunity, and inflammation, and as mentioned earlier, Nur77 can induce cell apoptosis by targeting mitochondria (Lith and de Vries, 2021). Studies have shown that *T. wilfordii* glycosides can interact with Nur77, thereby promoting its translocation from the nucleus to mitochondria. Within the mitochondria, Nur77 interacts with toll-like receptor 2, leading to its ubiquitination. The ubiquitinated Nur77 remains within the mitochondria, rendering these organelles sensitive to autophagy and facilitating the clearance of damaged mitochondria, thereby exerting anti-inflammatory effects (Sommer et al., 2021). Furthermore, glycosides of *T. wilfordii* have been shown to reduce the expression of CD80 and CD86 induced by IFN-γ and inhibit the expression of IL-23 and IL-1 in dendritic cells and T helper 1 cells (Wangchuk et al., 2018), thereby retarding the progression of inflammatory diseases. Furthermore, these glycosides have also been reported to inhibit RA-induced cell autophagy by suppressing activation of the toll-like receptor 2/high-mobility group protein signaling pathway, (Abdin and Hasby, 2014; Elshabrawy et al., 2017).

### 4.2 *Tripterygium wilfordii* and its derivatives inhibit the progression of RA via immune cell modulation

Normal functioning of the immune system is essential for maintaining bodily homeostasis; consequently, immune dysregulation can have particularly detrimental effects. Many autoimmune diseases develop as a response to abnormal immune expression, in which the body’s own tissues are mistakenly targeted, thus leading to the development of diseases such as RA, SLE, and psoriasis (Perl, 2017). In this context, *T. wilfordii* has been demonstrated to inhibit the proliferation of T and B cells, reduce the production of proinflammatory cytokines, and induce the apoptosis of activated lymphocytes (Lu et al., 2017). *T. wilfordii* glycosides have been shown to downregulate expression of the cell factors TNF-β, IL-6, IL-1, and IL-8 and upregulate the expression of IL-10, thereby inhibiting the activity of Th1 cells and promoting the transformation of Th1 to Th2 cells. These glycosides can also inhibit the synthesis and release of NF-κB, promote apoptosis, and reduce the occurrence and progression of immune reactions (Lin et al., 2001). In addition to these effects, *T. wilfordii* glycosides can contribute to lowering the levels of immunoglobulins IgE and IgA, enhance the function of CD8^+^ T cells, and inhibit the function of CD4^+^ T cells, thereby reducing the CD4^+^/CD8^+^ T cell ratio and inhibiting further development of abnormal immune responses (Liu et al., 2019).

The immunosuppressive effects *T. wilfordii* glycosides can be attributed to an inhibition of STAT3 phosphorylation, thereby suppressing Th17 cell function. Whereas Th17 cells promote inflammation, Tregs have anti-inflammatory functions (Balkrishna et al., 2020); therefore, maintaining an appropriate balance between Th17 cells and Tregs is essential for the maintenance of immune homeostasis. In this regard, it has been found that *T. wilfordii* can inhibit the expression of mammalian target of rapamycin, hypoxia-inducible factor 1-alpha, c-Myc, and protein kinase B, leading to metabolic blockade of Th17 cells and thus inhibiting their differentiation (Tang et al., 2020). Conversely, by upregulating carnitine palmitoyltransferase 1A and adenosine 5ʹ-monophosphate-activated protein kinase alpha, *T. wilfordii* promotes lipid fatty acid oxidation, thereby facilitating the generation of inducible Tregs (Marks, 2011). Moreover, it has been demonstrated that the administration of *T. wilfordii* significantly increases the Bax/Bcl-2 ratio, induces the release of mitochondrial cytochrome *C* into the cytoplasm, and activates caspase-3 and caspase-9, ultimately inducing T cell apoptosis via the mitochondrial apoptosis pathway (Jin et al., 2022).

Assisting cells, particularly dendritic cells, are professional antigen-presenting cells that have been established to be further targets of *T. wilfordii* glycosides (Zhang G. et al., 2013). These glycosides inhibit the differentiation, maturation, trafficking, and functioning of immature dendritic cells (Wangchuk et al., 2018). High concentrations (>20 ng/mL) of *T. wilfordii* glycosides have been observed to induce dendritic cell apoptosis via the sequential phosphorylation of p38 mitogen-activated protein kinase and activation of caspase-3 (Ziaei and Halaby, 2016). The chemotaxis of neutrophils and T cells mediated by dendritic cells has also been shown to be inhibited by a *T. wilfordii* glycoside-mediated reduction in STAT3 phosphorylation and activation of NF-κB (Zhang and Ma, 2010). Moreover, these glycosides prevent the differentiation of immature monocyte-derived dendritic cells by downregulating the expression of CD1a, CD40, CD80, CD86, and HLA-DR, as well as reduce the capacity of monocyte-derived dendritic cells to stimulate lymphocyte proliferation in allogeneic mixed lymphocyte reactions (Zhao et al., 2021). Recently, it has also been demonstrated that *T. wilfordii* glycosides inhibit the expression of IL-12/IL-23 in antigen-presenting cells by binding with CCAAT-enhancer-binding protein α, thus providing mechanistic insights into the immunomodulatory properties of these glycosides (Ziaei and Halaby, 2016).

### 4.3 *Tripterygium wilfordii* and its derivatives alleviate joint damage in RA

With respect to the treatment of RA, *T. wilfordii* glycosides have been demonstrated to inhibit the release of chemotactic factors from macrophages, thereby reducing their effects on synovial cells and chondrocytes, and thus inhibiting the abnormal proliferation of synovial cells (Baoqi et al., 2021). Furthermore, treatment with LLDT-8 has been found to suppress matrix metalloproteinase-13 production via the OPG/RANK/RANKL signaling pathway, increase OPG/RANKL expression, and attenuate collagen-induced arthritis (Shen et al., 2015). Moreover, *T. wilfordii-*derived triptolide can inhibit the expression of vascular endothelial growth factor (VEGF), VEGF receptor 1, and chemokine receptor 5 in the joint tissues of rats with adjuvant-induced arthritis. This activity has the effects of reducing the infiltration of lymphocytes and peripheral blood mononuclear cells into the synovium, thereby suppressing damage and degradation of the synovial tissue. Triptolide also targets neutrophils in mice with RA, promotes neutrophil apoptosis, and inhibits neutrophil autophagy and neutrophil extracellular trap formation, thereby reducing tissue damage and inflammation (Zhang B. et al., 2013). Studies have also shown that *T. wilfordii* glycosides can significantly alleviate paw swelling in rats (as determined by performing traditional anti-inflammatory tests), downregulate the expression of VEGF and VEGF receptor 2, and reduce vascular permeability and angiogenesis (Ashrafizadeh et al., 2022). Furthermore, by inhibiting the expression of *miR-4478*, *T. wilfordii* upregulates WAKMAR2, which in turn promotes an upregulation of E2F transcription factor 1 and p53 expression, with the outcome of this cascade being a suppression of fibroblast-like synoviocyte expression (Zhou et al., 2021).

## 5 *Tripterygium wilfordii* treatment of other rheumatic diseases

### 5.1 Systemic lupus erythematosus

SLE is a chronic autoimmune disease characterized by a wide range of clinical symptoms, notably the abnormal production of self-antibodies, such as antinuclear and anti-double-stranded DNA antibodies (Moulton et al., 2017). SLE predominantly affects women of childbearing age, with a male-to-female patient ratio of approximately 1:9, and is accordingly speculated to be influenced by female hormones (Ameer et al., 2022). Moreover, genetic and environmental factors have been established to contribute to the development of SLE (Li et al., 2010). *T. wilfordii* has a long history of use in the treatment of SLE and, when administered with appropriate care, patients can experience satisfactory alleviation. In therapeutic strategies for this disease, the use of selected immunosuppressive agents can contribute to reducing the cumulative steroid dose and the prevention of disease relapse. Similarly, in patients with refractory or relapsed SLE, the use of other immunosuppressive agents can contribute to a reduction in steroid dosage, control of disease activity, and improvements in the rates of clinical remission (Li and Yang, 2022). In a study of patients with SLE, treatment with a *T. wilfordii* extract combined with prednisone was demonstrated to increase the levels of CD4^+^ and CD25^+^ T cells, thus enhancing immune tolerance in these patients. Based on the findings of these studies, it can be concluded that the regulatory effects of *T. wilfordii* extract on T cells further enhance its protective effects in patients with SLE. Studies have shown that neutrophil extracellular traps, web-like structures released by neutrophils, are involved in driving autoimmunity and tissue damage in SLE (Georgakis et al., 2021), and in this regard, it has been found that *T. wilfordii* can downregulate the activation of spleen tyrosine kinase and phosphorylation of mitogen-activated protein kinase, extracellular signal-regulated kinase, and IκBα, as well as the guanine methylation of histones. By downregulating the spleen tyrosine kinase–mitogen-activated protein kinase kinase–extracellular signal-regulated kinase–NF-κB signaling cascade, *T. wilfordii* can effectively inhibit neutrophil oxidative bursts and the formation of neutrophil extracellular traps induced by diverse inflammatory stimuli, thereby inhibiting SLE development (Kato et al., 2013).

### 5.2 Psoriasis

Psoriasis, a chronic skin disease affecting over 100 million people worldwide (Griffiths and Barker, 2007), is characterized by the typical symptoms of erythema, itching, and scaling, leading to physical discomfort and psychological burden (Kamiya et al., 2019). Psoriasis is a disease involving both the innate and adaptive immune systems, in which keratinocytes, dendritic cells, and T cells play key roles in disease development (Li and Yang, 2022). It has been found that an extract of *T. wilfordii* can inhibit the excessive proliferation of human keratinocytes (HaCaT cells) and significantly reduce the mRNA levels of inflammatory cytokines such as *TNF-α* and *IL-6* in HaCaT cells. By modulating the interactions between keratinocytes and downstream dendritic cells and T cells in the immune system, as well as reducing the expression levels of inflammatory cytokines in the skin and circulation, *T. wilfordii* has an inhibitory effect on systemic inflammation, thus achieving therapeutic effects in patients with psoriasis (Parveen et al., 2019). Furthermore, *T. wilfordii* extract has been shown to regulate the activity of dendritic cells and T cells, suppress the generation of CD4 T and Th17 cells, inhibit the development of Th17 cells, and inhibit the proliferation of keratinocytes and epidermal cells, as well as the expression of inflammatory cytokines such as TNF-α and IL-17A (Guo et al., 2020). Similarly, in murine animal models, treatment with *T. wilfordii* extract has been shown to significantly reduce white scales, erythema, inflammatory cells, excessive keratinization, and incomplete keratinization. Furthermore, this treatment contributes to marked reductions in the release of inflammatory factors, such as IL-17, INF-γ, and IL-22, in the plasma at the mRNA and protein levels (Maqbool et al., 2021).

### 5.3 Crohn’s disease

Crohn’s disease is a chronic granulomatous inflammatory condition that affects the entire gastrointestinal tract, particularly the ileocecal and perianal areas (Baumgart and Sandborn, 2012). Although the exact etiology of Crohn’s has yet to be sufficiently established, several specific factors are known to be directly associated with the disease, including genetic factors, intestinal immune dysregulation, and alterations in the gut microbiota (Mazal, 2014). Recent findings have emphasized the key role played by the gut microbiota in the development of this disease, which is in turn, closely associated with sustained adverse inflammatory responses (Rodríguez et al., 2020). Currently, the treatment options for patients with CD are rather limited, although the use of natural products, such as extracts of *T. wilfordii*, which may have fewer side effects than conventional medications, has clear appeal (Zhang et al., 2020). Studies have reported that an extract of *T. wilfordii* induces apoptosis in T lymphocytes and dendritic cells by inhibiting the NF-κB signaling pathway and reduces the production of pro-inflammatory cytokines such as TNF-α and IL-1, thereby alleviating intestinal inflammation in patients (Ren et al., 2013). It has also been found that FoxP3 Tregs play a vital role in maintaining intestinal immune balance via the actions of IL-10 and transforming growth factor-beta, and that *T. wilfordii* extract promotes mucosal healing by upregulating FoxP3 Tregs and IL-10, downregulating inflammatory cytokines such as TNF-α, and modulating the local production of other inflammatory cytokines (Li et al., 2014).

### 5.4 Multiple sclerosis

**FIGURE 3:**
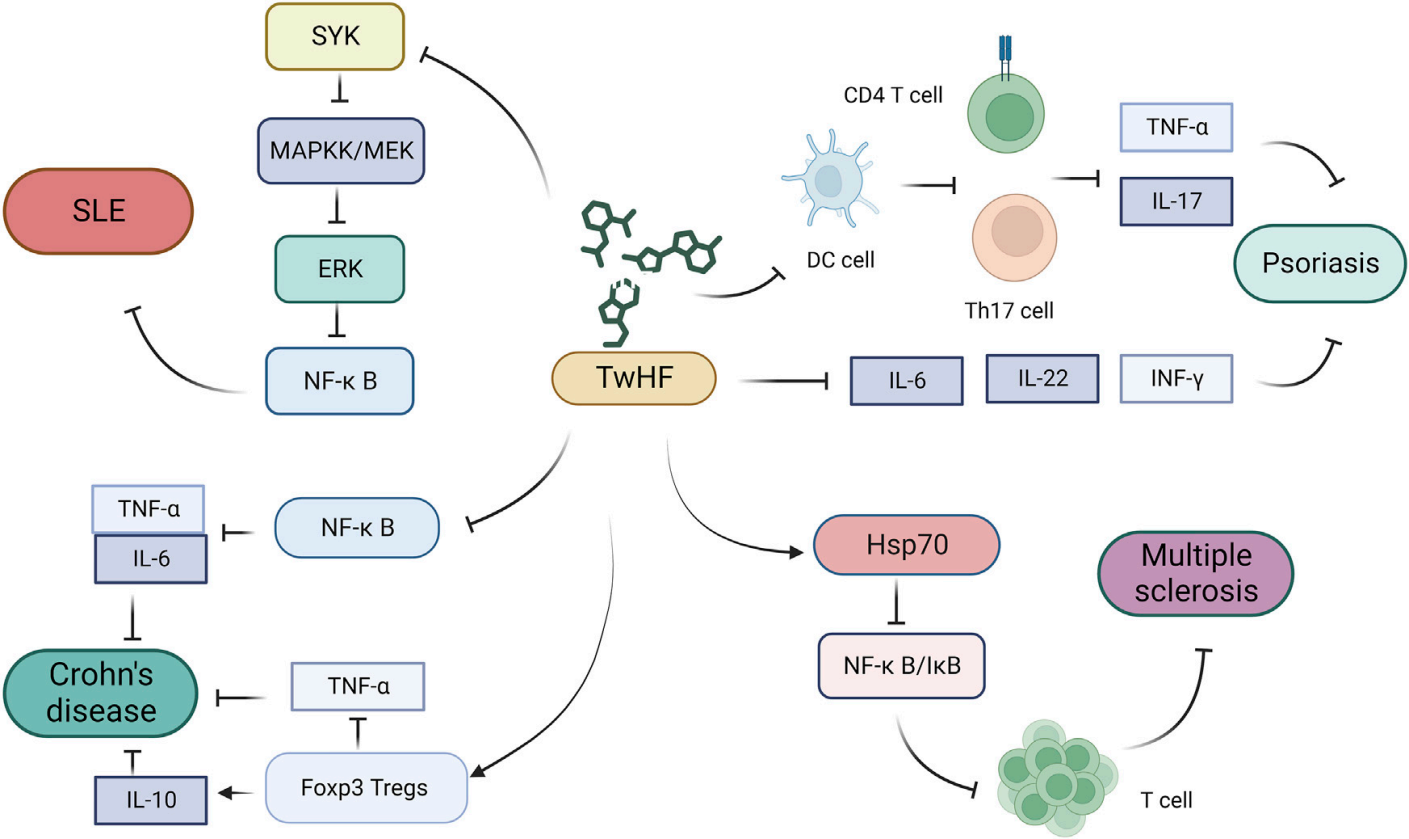
Mechanisms underlying the efficacy of *Tripterygium wilfordii* in the treatment of other rheumatic diseases (Ma et al., 2020). *T. wilfordii* can downregulate the activation of SYK, exerting an inhibitory effect on the pathogenesis of SLE through downregulation of the SYK–MEK–ERK–NF-κB signaling cascade (Schwenzer et al., 2010). *T. wilfordii* suppresses the proliferation of CD4^+^T and Th17 cells, diminishes the expression of inflammatory cytokines, such as TNF-α and IL-17A, and further inhibits the release of inflammatory factors, including IL-6, INF-γ, and IL-22. Consequently, this attenuates the progression of psoriasis (Tsitrouli et al., 2021). *T. wilfordii* inhibits the NF-κB signaling pathway, leading to reduced production of the pro-inflammatory cytokines TNF-α and ILN-6, thereby alleviating intestinal inflammatory responses in patients with Crohn’s disease. Additionally, *T. wilfordii* upregulates FoxP3 Tregs and IL-10 expression while downregulating the inflammatory cytokine TNF-α, thereby suppressing the progression of Crohn’s disease (Shi et al., 2020). *T. wilfordii* elevates Hsp70 levels to inhibit the NF-κB/IκB complex, thereby attenuating T cell proliferation and differentiation, resulting in the suppression of inflammatory responses in multiple sclerosis. (SYK, spleen tyrosine kinase; MAPKK/MEK, mitogen-activated protein kinase; ERK, extracellular signal-regulated kinase; NF-κB, nuclear factor kappa-B; SLE, systemic lupus erythematosus; DC, dendritic cell; Th17, T helper cell 17; IL-17, interleukin 17; IL-6, interleukin 6; IL-10, interleukin 10; IL-22, interleukin 22; IFN-γ, gamma interferon; TNF-α, tumor necrosis factor alpha; FoxP3, forkhead box Protein 3; IκB, inhibitor of NF-κB; Hsp70, heat shock protein 70).

Multiple sclerosis, also known as demyelinating disease, is a multifocal chronic autoimmune inflammatory disease of the central nervous system (Doshi and Chataway, 2016). Although the etiology of multiple sclerosis has yet to be elucidated, the accumulation and activation of monocytes within the central nervous system have been established to play key roles in its pathogenesis. Chemokines are major players in leukocyte recruitment and activation at the sites of inflammation (Moreira et al., 2006), and studies have shown that neuroinflammation can have both detrimental and beneficial effects on the function of neurons and glial cells, with the NF-κB signaling pathway playing a pivotal role in controlling this process. These findings accordingly indicate that selective anti-NF-κB treatment strategies may contribute to minimizing damage during the acute and chronic inflammatory phases of this disease (Webb et al., 2017; Sanadgol et al., 2018). Experimental autoimmune encephalomyelitis is a T cell-mediated autoimmune disease of the central nervous system that serves as an animal model of human multiple sclerosis (Baecher-Allan et al., 2018), and it has been proposed that *T. wilfordii* may act as an active suppressor of experimental autoimmune encephalomyelitis, whereas LLDT-8 has been shown to inhibit T cell proliferation and activation (Fu et al., 2006). Specifically, it can attenuate the inflammatory response in the experimental autoimmune encephalomyelitis model by increasing the levels of heat shock protein 70 and stabilizing the NF-κB/IκB complex (Figure 3) (Zheng et al., 2013).

## 6 Toxic effects of *Tripterygium wilfordii*

In addition to its therapeutic efficacy, the safety of the medicinal use of *T. wilfordii* has been extensively studied. Although preparations of this plant are generally well tolerated, they can cause a rage of side effects, including gastrointestinal discomfort, rashes, alopecia, liver and kidney damage, reproductive toxicity, and myelosuppression (Xi et al., 2017). A recent meta-analysis of *T. wilfordii* in the treatment of renal disease indicated that approximately 11.7% of patients experienced adverse events after taking the *T. wilfordii* preparations. Briefly, the authors found that 273 studies reported gastrointestinal reactions, in which 4,843 (8.6%) patients had elevated levels of alanine transaminase (twice the normal level), 12.7% of female patients had menstrual disorders, approximately 4.9% of patients experienced cardiovascular events, and approximately 6.5% and 7.8% of patients experienced hematological and mucosal damage, respectively. Moreover, 43 studies reported renal damage in 193 patients and 159 studies reported alopecia, weight loss, and fatigue in 1,007 patients (Zhang et al., 2016). However, despite their potential severity, these adverse effects are generally reversible and can be prevented through careful monitoring and dose adjustment (Baoqi et al., 2021).

### 6.1 Hepatotoxic side effects

During its hepatic metabolism, metabolites of *T. wilfordii* undergo lipid peroxidation, which can lead to hepatocytic necrosis. Hepatotoxicity is among the major toxic reactions associated with the use of *T. wilfordii*, which manifests clinically as liver discomfort, hepatomegaly, and abnormal liver function. This is primarily characterized by elevated activities of alanine aminotransferase and aspartate aminotransferase, increased levels of total bile acids and total bilirubin, lower levels of prealbumin expression, hepatocellular steatosis, necrosis, and inflammatory cell infiltration (Zhong et al., 2013). Mechanistic studies that have examined the hepatotoxicity of *T. wilfordii* glycosides have revealed that when administered at high doses, these glycosides can inhibit the mRNA expression of key hepatic cytochrome P450 family members, including *CYP27A1* and *CYP8B1*, as well as that of antioxidative enzymes such as SOD-1 and glutathione peroxidase 1. The consequent reduced protein expression of these enzymes results in bile stasis, mitochondrial damage, and a series of oxidative stress responses within the liver (Li et al., 2020).

### 6.2 Nephrotoxic side effects

The nephrotoxicity associated with *T. wilfordii* usage is mainly attributable to its diterpenoid, triterpenoid, and alkaloid metabolites, which can damage renal tubular epithelial cells. The clinical manifestations of *T. wilfordii*-induced nephrotoxicity include oliguria, edema, hematuria, hypotension, and hyperkalemia (Zhang Q. et al., 2018). Administration of a *T. wilfordii* extract to rats through gavage has be shown to result in elevated levels of blood creatinine, blood urea nitrogen, and uric acid, as well as causing renal glomerular atrophy, liquefaction, and necrosis of the local renal tubules (Shen et al., 2020). Further investigations have revealed that the molecular mechanisms underlying *T. wilfordii*-induced nephrotoxicity may involve an increase in the levels of the death receptor Fas and its ligand FasL through the mediation of triptolide. Additionally, cytochrome *C*, an intrinsic pathway mediator, is released into the cytoplasm, leading to alterations in electron transport, loss of mitochondrial membrane potential, and induction of apoptosis through multiple pathways (Feng et al., 2019). It has also been suggested that *T. wilfordii*-induced renal injury may be attributable to an impairment of angiotensin II receptor synthesis, resulting in elevated levels of ROS in renal tissue and the promotion of cell apoptosis by damaging the renal glomeruli (Zhang C. et al., 2018).

### 6.3 Reproductive toxicity

Toxic side effects of *T. wilfordii* have also been reported for the reproductive system, as exemplified by instances of amenorrhea in women and reduced sperm counts in men (Singla and Challana, 2014), and these have been found to be significantly correlated with patient age, drug dosage, and course of treatment. As a cytotoxic medication, *T. wilfordii* mainly acts on follicular cells, thereby reducing the level of estrogen secreted by these follicles (Deng et al., 2022). Additionally, preparations of this plant can reduce progesterone levels, thereby altering the levels of hormones in the hypothalamus–pituitary–gonadal axis and leading to abnormalities in the menstrual cycle and thus menstrual disorders or amenorrhea. Furthermore, *T. wilfordii* has been shown to reduce the expression of spermatogenesis-related genes and inhibit the morphological and functional development of spermatozoa, leading to reduced fertility (Ruan et al., 2018), whereas in animal experiments, *T. wilfordii* has been found to induce mitochondrial apoptosis of ovarian granulosa cells in NIH mice, leading to ovarian damage (Zeng et al., 2017). In the latter case, however, it was established that the perturbation of ovarian function caused by tretinoin was reversible, and that function recovered naturally after discontinuing administration of the drug.

### 6.4 Gastrointestinal side effects

Adverse reactions of the human gastrointestinal tract associated with the oral administration of different preparations of *T. wilfordii* primarily include a loss of appetite, dry mouth, nausea and vomiting, constipation, abdominal pain, and diarrhea (Liu et al., 2018). However, most patients tolerate these symptoms and recover after discontinuing administration of the drug. In laboratory studies, mice administered the LD_50_ dosage of triptolide, either intraperitoneally and orally, showed significant congestion at the base of the stomach and irregularly scattered intestinal ulcers. In a study on the *in vivo* activity of triptolide, it was found that although this compound was readily absorbed by the gastrointestinal tract, the absorption was incomplete, which may account for the gastrointestinal tract irritation associated with triptolide usage (Liu Y. et al., 2013).

### 6.5 Other side effects

Abnormal skin-related symptoms are an additional frequently reported side effect of *T. wilfordii* usage, among which skin pigmentation, drug eruption, erythema nodosum, oral mucosal herpes, and skin allergic vasculitis are common reactions (Tao et al., 2002). However, most of these responses require no special treatment and resolve after a reduction in dosage or discontinued administration of the drug. Furthermore, celastrol has been shown to inhibit cell viability and migration, block the cell cycle at the G0/G1 phase, promote apoptosis by enhancing the expression of caspase-3 and Bax, and reduce the expression of Bcl-2 in human biliary epithelial cell death, thereby inducing cholangiocytotoxicity (Y-JJCT and Drugs, 2020). Furthermore, triptolide has been found to inhibit the proliferation and viability of inner ear stem cells, and induces apoptosis by enhancing the expression of the DNA damage repair proteins γH2AX and 53BP1. Moreover, it has been speculated that triptolide-induced inner ear stem cell cytotoxicity may be associated with mitochondrial dysfunction caused by optic nerve atrophy and incision (Tang et al., 2019).

## 7 Discussion

*T. wilfordii* has a long-standing history and extensive application in the treatment of rheumatic and autoimmune diseases, often with notable clinical efficacy. With continual advances in clinical research and progress in the pharmacology and toxicology of *T. wilfordii*, a diverse range of bioactive metabolites within this plant have gradually been discovered and demonstrated to have notable anti-inflammatory and immunomodulatory effects. In this review, we have systematically summarized the historical evolution of *T. wilfordii* in the treatment of rheumatic and autoimmune diseases, along with its global distribution. We also describe the effective metabolites produced by this plant and their derivatives, which have been shown to have notable anti-inflammatory, anticancer, immunosuppressive, and immunomodulatory properties (Table 3). Additionally, we present a comprehensive overview of the mechanistic actions of *T. wilfordii* in the treatment rheumatoid arthritis, including immune suppressive, anti-inflammatory, anti-angiogenic, and bone and cartilage protective effects, and discuss the mechanisms underlying the efficacy of *T. wilfordii* treatment for other rheumatic and autoimmune diseases, namely, SLE, psoriasis, Crohn’s disease, and multiple sclerosis. Moreover, we highlight the potential toxic effects of *T. wilfordii* preparations, primarily in terms of hepatorenal damage. However, although these effects have been observed in clinical settings and are clearly undesirable, many eventually resolve without intervention, or can be partially reversed or alleviated through dose adjustment.

**TABLE 3:** Preclinical research on *Tripterygium wilfordii* Hook F.

Disease	Properties	Related pathways and molecules
RA	Anti-inflammatory	ROS-NF-κB-NLRP3
	Immune regulation	IκB- IKKα/IKKβ-P65
	Alleviate joint damage	TNF、IFN、IL-6、IL-8、
		STAT3-Mtor/Akt-Th17 p38 MAPK/caspase3
		OPG/RANK/RANKL
		VEGF/FLT1/CCR5
SLE	Anti-inflammatory	SYK-MEK-ERK-NF-κB
Psoriasis	Anti-inflammatory	IL-6, INF-γ, and IL-22
	Immune regulation	DCs-CD4^+^T/Th17- TNF-α/IL-17A
Crohn’s disease	Anti-inflammatory	NF-κB- TNF-α/IL-6
		TNF-α- Foxp3 Tregs- IL-10
Multiple sclerosis	Anti-inflammatory	Hsp70- NF-κB/IκB
	Immune cell regulation	

Although significant progress has been made in the treatment of rheumatic and autoimmune diseases using *T. wilfordii*, particularly in the case of RA, limitations persist in advanced chemical and pharmacological methods, as well as in the accumulation of experience in clinical practice. Despite substantial accomplishments in clinical trials, meta-analyses, experimental studies, and guideline development, gaps remain in our understanding of the pathogenesis and etiology of rheumatic and autoimmune diseases, as well as the precise mechanisms of action of *T. wilfordii* preparations. Currently, these gaps present a substantial hurdle hampering the more widespread application of *T. wilfordii* preparations in countries other than China, as well as the realization of personalized treatment for patients. Moreover, *T. wilfordii* and the extracts thereof contain a diverse range of metabolites that may have synergistic or antagonistic effects, which consequently presents considerable challenges in establishing clear associations between these metabolites and their corresponding biological targets. Accordingly, elucidating the potential molecular mechanisms underlying the effects of *T. wilfordii* metabolites and delineating their functional and toxicological characteristics are of paramount importance. An integrated approach that combines clinical data with systems biology, network pharmacology, analytical chemistry, and molecular biology approaches is necessary to achieve these goals.

## Figures and Tables

**Table:** 

***T. wilfordii***	Tripterygium wilfordii Hook F
**NSAIDs**	nonsteroidal anti-inflammatory drugs
**DMARDs**	disease-modifying antirheumatic drugs
**PARP**	poly ADP-ribose polymerase
**NF-κB**	nuclear factor kappa-B
**TLR4**	toll like receptor 4
**RANKL**	receptor activator of nuclear factor-κ B ligand
**RANK**	receptor activator of nuclear factor-κ B
**OPG**	osteoprotegerin
**CIA**	collagen-induced arthritis
**SQSTM1**	sequestosome1
**WA**	Wilforlide A
**MCP1**	monocyte chemoattractant protein-1
**GM-CSF**	granulocyte-macrophage colony stimulating factor
**M-CSF**	macrophagecolony stimulating factor
**LPS**	Lipopolysaccharides
**IFN-γ**	interferon gamma
**HIV**	human immunodeficiency virus
**\MMP-9**	matrix metalloproteinase-9
**RA,IL-6**	interleukin 6
**IL-10**	interleukin 10
**IL-17**	interleukin 17
**IL-18**	interleukin 18
**TNF-α**	tumor necrosis factor α
**iNOS**	inducible nitric oxide synthase
**COX-2**	cyclooxygenase-2
**PGE2**	prostaglandin E2
**NO**	nitric oxide
**LLDT-8**	5R-hydroxytriptolide
**lncRNA**	long non-coding RNA
**GBM**	glomerular basement membrane
**STAT1**	signal transducerand activator of transcription 1
**IRF-1**	interferon regulatory factor-1
**IL-2**	interleukin 2
**SCD1**	stearoyl-CoA desaturase-1
**PPARα**	peroxisome proliferator-activated receptor alpha
**RCTs**	randomized controlled trials
**MTX**	methotrexate
**TNF-β**	tumor necrosis factor β
**NLRP3**	NOD-like receptor protein 3
**FCA**	Freund’s adjuvant
**THP-1**	tohoku hospital pediatrics-1
**SOD**	superoxide dismutase
**ROS**	reactive oxygen species
**RNS**	reactive nitrogen species
**GSH-Px**	glutathione peroxidase
**MCP-1**	monocyte chemoattractant protein-1
**HRPE**	human retinal pigment epithelial
**ARPE-19**	adult retinal pigment epithelial cell line-19
**IKK**	inhibitor of kappa B kinase
**IκBα**	inhibitor of NF-κB
**FoxP3**	forkhead box prote 1
**ET-1**	endothelin-1
**HMGB1**	high mobility group protein 1
**DCs**	dendritic cells
**TLR2**	toll-like receptors 2
**SLE**	systemic lupus erythematosus
**Tregs**	regulatory T cells
**APCs**	antigen-presenting cells
**FAO**	fatty acid oxidation
**CPT1A**	carnitine palmitoyltransferase 1A
**AMPKα**	adenosine 5‘-monophosphate-activated protein kinase α
**MAPK**	mitogen-activated protein kinase
**MoDCs**	monocyte-derived DCs
**VEGF**	vascular endothelial growth factor
**FLT1**	vascular endothelial growth factor receptor 1
**CCR5**	chemokine receptor 5
**PBMCs**	peripheral blood mononuclear cells
**VEGFR2**	vascular endothelial growth factor receptor 2
**NETs**	neutrophil extracellular traps
**SYK**	spleen tyrosine kinase
**ERK**	extracellular signal-regulated kinase
**TGF-β**	transforming growth factor-beta
**MS**	multiple sclerosis
**CNS**	central nervous system
**EAE**	experimental autoimmune encephalomyelitis
**HSP70**	heatshockprotein70
**ALT**	alanine aminotransferase
**AST**	aspartate aminotransferase
**TBA**	total bile acids
**TBIL**	total bilirubin
**E2F1**	E2F transcription factor 1
**FLS**	fibroblast-like synoviocyte
